# Dasatinib vs. imatinib in patients with chronic myeloid leukemia in chronic phase (CML-CP) who have not achieved an optimal response to 3 months of imatinib therapy: the DASCERN randomized study

**DOI:** 10.1038/s41375-020-0805-1

**Published:** 2020-04-07

**Authors:** Jorge E. Cortes, Qian Jiang, Jianxiang Wang, Jianyu Weng, Huanling Zhu, Xiaoli Liu, Andreas Hochhaus, Dong-Wook Kim, Jerald Radich, Michael Savona, Patricia Martin-Regueira, Oumar Sy, Renuka Gurnani, Giuseppe Saglio

**Affiliations:** 1 Georgia Cancer Center, Augusta University, Augusta, GA USA; 2 Peking University People’s Hospital, Beijing, China; 3 Institute of Hematology and Blood Diseases Hospital, Chinese Academy of Medical Sciences, Tianjin, China; 4 Guangdong Provincial People’s Hospital, Guangdong Academy of Medical Sciences, Guangzhou, Guangdong China; 5 West China Hospital of Sichuan University, Chengdu, Sichuan China; 6 Nanfang Hospital, Southern Medical University, Guangzhou, Guangdong China; 7 Universitätsklinikum Jena, Jena, Germany; 8 The Catholic University of Korea, Seoul, Republic of Korea; 9 Fred Hutchinson Cancer Research Center, Seattle, WA USA; 10 Vanderbilt-Ingram Cancer Center, Vanderbilt University School of Medicine, Nashville, TN USA; 11 Bristol Myers Squibb, Princeton, NJ USA; 12 University of Turin, Turin, Italy

**Keywords:** Combination drug therapy, Epidemiology

## Abstract

Early molecular response is associated with improved probability of deep molecular response and superior survival in patients with CML-CP. However, ~1 in 3 patients on first-line imatinib do not achieve this threshold. The phase 2b DASCERN trial (NCT01593254) assessed the outcome of early switch to dasatinib in patients with suboptimal response to first-line imatinib. Adult patients with CML-CP were randomized (2:1) to receive 100 mg dasatinib (*n* = 174) or continue imatinib at ≥400 mg (*n* = 86). The primary endpoint was the rate of major molecular response (MMR) at 12 months, which was 29% (dasatinib) and 13% (imatinib; *P* = 0.005). After ≥2 years of follow-up, 45 patients (52%) randomized to continue imatinib had crossed over to dasatinib. Considering treatment crossover, the 2-year cumulative MMR rate was 64% with dasatinib and 41% with imatinib (66% and 67%, respectively by intent-to-treat). Adverse events were consistent with the established safety profiles of both drugs. The results of this first prospective study support early monitoring of patients treated with first-line imatinib, and suggest that switching to dasatinib in cases of suboptimal response may offer clinical benefit. Further follow-up is needed to assess the long-term clinical benefit of early switching.

## Introduction

Achieving early molecular response (EMR), defined as a reduction in *BCR-ABL1* transcripts to ≤10% (International Scale [IS]) at 3 or 6 months after initiating tyrosine kinase inhibitor (TKI) treatment, has been shown to improve the probability of achieving a subsequent deep molecular response (DMR; typically MR^4.5^ or *BCR-ABL1* ≤ 0.0032% [IS]) and to be associated with superior progression-free and overall survival (OS) in chronic myeloid leukemia in chronic phase (CML-CP) [1, 2]. The prognostic significance of EMR has been established for both imatinib and second-generation TKIs in the first-line setting. Patients treated with dasatinib or imatinib with EMR at 3 months in DASISION (the Dasatinib vs. Imatinib Study in Treatment-Naïve Chronic Myeloid Leukemia Patients Trial) had an increased likelihood of achieving complete cytogenetic response (CCyR), major molecular response (MMR), increased progression-free survival (PFS), and decreased likelihood of progression to CML in accelerated phase or blast crisis (CML-AP/BC) [2]. Similar improvement in long-term outcomes has also been reported with other second-generation TKIs [3, 4]. However, nearly one-third of patients with CML-CP treated with first-line imatinib fail to achieve EMR [2, 3] and, compared with imatinib, second-generation TKIs have been shown to be associated with a 96% reduction in the risk of a poor cytogenetic response at the 3-month timepoint [5].

In light of this growing body of evidence, the 2013 European LeukemiaNet (ELN) recommendations and the National Comprehensive Cancer Network Clinical Practice Guidelines (NCCN Guidelines®) now include EMR as a treatment milestone for patients with newly diagnosed CML-CP, and consider *BCR-ABL1* ≤ 10% (IS) at 3 months as the optimal molecular response [1, 6]. Although the ELN recommendations consider *BCR-ABL1* > 10% at 3 months a warning, a change in therapy is not conclusively recommended, reflecting the current lack of data from prospective clinical trials describing how an early change in therapy at this time may translate into a clinical benefit.

DASCERN (Study of Dasatinib vs. Imatinib in Patients With Chronic Myeloid Leukemia Who Did Not Have Favorable Response to Imatinib; NCT01593254) is the first and only prospective randomized trial to explore the potential benefit of an early switch to dasatinib in patients with lack of EMR to first-line imatinib. Here we present the first results of this study, including response rates and 2-year survival outcomes.

## Materials and methods

### Study design and eligibility

**Fig. 1:**
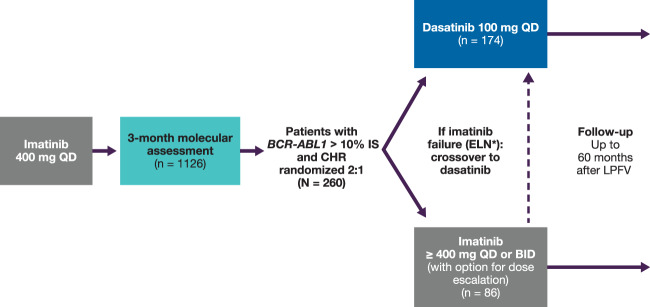
Study design. *Patients initially randomized to imatinib, meeting ELN 2013 failure criteria, and without dasatinib-resistant mutations, were crossed over to the dasatinib arm. BID twice daily, CHR complete hematologic response, ELN European LeukemiaNet, IS International Scale, LPFV last patient first visit, QD once daily.

DASCERN is an open-label, randomized, international, multicenter phase 2b trial of dasatinib vs. imatinib in patients with Philadelphia chromosome-positive (Ph+) CML-CP who had achieved complete hematologic response (CHR), but had *BCR-ABL1* > 10% (IS) 3 months after starting first-line treatment with imatinib 400 mg once daily (QD) (Fig. 1). Molecular response assessments prior to enrollment were performed at a central laboratory, and patients with *BCR-ABL1* ≤ 10% (IS) were ineligible. Patients with *BCR-ABL1* > 10% (IS) at 3 months were considered eligible and randomized 2:1 to receive dasatinib 100 mg QD (early switch) or continue on imatinib (at any dose selected by the enrolling investigator). Randomization occurred up to 8 weeks after the 3-month molecular assessment. Patients were randomized by means of an interactive voice response system, with randomization performed using permuted blocks within each stratum and stratified by Sokal score (high, intermediate, low, or unknown) and time between the 3-month molecular assessment and randomization (≤4 weeks vs. >4 weeks). Patients randomized to imatinib who subsequently met ELN 2013 criteria for treatment failure [7] were crossed over to dasatinib unless they had documented dasatinib-resistant *BCR-ABL* mutations (e.g., T315I/A, F317L, V299L) as assessed in a central laboratory. Assessment of prior mutations could be performed at local laboratories, but was not mandatory. Mutational analysis was performed following a suboptimal response, treatment failure, or progression, and at the end of treatment or prior to any change in therapy.

Sample size for randomized patients was computed based on the following assumptions: 2:1 randomization ratio, two-sided superiority test with *α* = 0.05 and 90% power, and MMR at 12 months of 10% for imatinib and 25% for dasatinib.

Eligible patients were aged ≥18 years and had started imatinib monotherapy within 6 months of the initial CML-CP diagnosis (PH+ or *BCR-ABL1* detection). Patients were required to be tolerating imatinib 400 QD (maximum of cumulative 2 weeks’ interruption within the prior 3 months permitted), and to have an Eastern Cooperative Oncology Group (ECOG) performance status 0–2, with adequate renal/hepatic function.

Patients could receive dasatinib or imatinib for up to 60 months after randomization of the last patient, or until disease progression, treatment failure, unacceptable toxicity, withdrawal of consent, or discontinuation of the study. All patients provided written informed consent in accordance with the Declaration of Helsinki and institutional guidelines before study entry. The study protocol was approved by institutional review boards (and/or ethics committee) of each participating center, as well as the competent national authority.

### Study endpoints

The primary endpoint in DASCERN was defined as the proportion of patients who achieved an MMR at 12 months after day 1 of first-line imatinib treatment in patients randomized at 3 months to dasatinib or imatinib (up to 9 months after randomization). Key secondary endpoints include time to MMR, time to MR^4.5^, PFS, and OS. Tertiary endpoints include development of *BCR-ABL1* mutations, safety and tolerability, molecular and cytogenetic response over time, and benefit of early switch to dasatinib (at 3 months) over a later switch/crossover at the time of imatinib failure (based on ELN 2013 failure criteria).

### Evaluations and study definitions

Data from all evaluations, except for the primary endpoint, were from a 24-month data cut. For all evaluations, all patients were followed every 3 months for the first 24 months, then every 6 months until month 60; patients were then followed annually. Safety assessments and molecular analyses were conducted at month 4 or 5, and 6, and then every 3 months for up to 24 months. For patients continuing their assigned treatment beyond 24 months, safety assessments, as well as hematology and molecular analysis/quantitative polymerase chain reaction (qPCR) were conducted every 6 months, and cytogenetic assessments (conventional or fluorescence in situ hybridization [FISH; peripheral blood]) were conducted every year. A cytogenetic response was based on the prevalence of Ph+ cells in metaphase in bone marrow according to standard criteria (CCyR = 0% Ph+ cells) [7]. MR^4.5^ was considered ≤0.0032% *BCR-ABL1* (IS). Time to MMR or MR^4.5^ was defined as the time from randomization until first PCR showing MMR or MR^4.5^. PFS was defined as time from randomization to transformation to CML-AP/BC or death from any cause during treatment. OS was defined as the time between the randomization date and death date. Adverse events (AEs) and serious AEs were assessed according to the National Cancer Institute’s Common Terminology Criteria for Adverse Events version 4.0 [8].

### Statistical analysis

The primary endpoint analysis was performed using the Cochran–Mantel–Haenszel (CMH) test [9], stratified by Sokal score and time from molecular analysis to randomization. The intent-to-treat (ITT) population included all patients initially randomized to each arm, irrespective of crossover. An exact 95% confidence interval (CI) for the difference in MMR rate at 12 months was computed. Time-to-event endpoints were estimated using the Kaplan–Meier, Brookmeyer–Crowley, and Fine and Gray methods (calculation of competing risk), and compared between treatment groups using a two-sided stratified log-rank test [10–14]. Competing risks for cumulative incidence of MMR were death or bone marrow transplantation. Patients who did not achieve MMR or MR^4.5^ were censored at their last molecular assessment date. Differences in response rates were assessed using the CMH test. For primary endpoint analyses, any patient with treatment failure after randomization who discontinued from the study (any arm, for any reason) or crossed over to dasatinib from the imatinib arm was considered a nonresponder. A sensitivity analysis of PFS and OS was performed on randomized patients where patients who crossed over to dasatinib after failure on imatinib were censored at the date of crossover.

## Results

### Baseline patient characteristics and patient disposition

A total of 1126 patients were enrolled in the study from September 12, 2012, to November 8, 2016, of whom 260 patients with *BCR-ABL1* > 10% were randomized (dasatinib, *n* = 174; imatinib, *n* = 86). Baseline patient characteristics for all randomized patients are shown in Table 1. Median age was 37 years (range 18–82) and 248 (95%) patients were <65 years old. Sokal scores were evenly distributed (low, 28%; intermediate, 30%; high, 24%; unknown, 18%). Patients were predominantly male (78%) and Asian (73%), and most (84%) had an ECOG performance status of zero. All patients had b2a2 (e13a2) or b3a2 (e14a2) transcripts.

**Table 1:** Baseline patient characteristics.

	Dasatinib (*n* = 174)	Imatinib (*n* = 86)	Total (*N* = 260)
Age, median (range), years	35 (18–82)	40 (18–73)	37 (18–82)
Age categorization			
<65 years	166 (95)	82 (95)	248 (95)
≥65 years	8 (5)	4 (5)	12 (5)
Male	133 (76)	70 (81)	203 (78)
Race			
White	36 (21)	15 (17)	51 (20)
Black or African American	4 (2)	3 (4)	7 (3)
Asian	127 (73)	63 (73)	190 (73)
Other	7 (4)	5 (6)	12 (5)
Sokal score			
Low	47 (27)	26 (30)	73 (28)
Intermediate	51 (29)	26 (30)	77 (30)
High	44 (25)	19 (22)	63 (24)
Unknown	32 (18)	15 (17)	47 (18)
ECOG performance status			
0	142 (82)	75 (87)	217 (84)
1	27 (16)	10 (12)	37 (14)
2	0	1 (1)	1 (<1)
Not reported	5 (3)	0	5 (2)

Values are *n* (%) unless otherwise noted.

*ECOG* Eastern Cooperative Oncology Group.

After a minimum of 24 months’ follow-up, 135 (79%) patients randomized to dasatinib and 68 (79%) patients randomized to imatinib (three patients randomized to dasatinib were not treated) were continuing on treatment (Table 2 and Supplementary Fig. S1). Of the 45 patients randomized to imatinib who crossed over to dasatinib, 32 (71%) patients continued to receive dasatinib therapy at 24 months. The median daily dose was 100 mg (range 26–136) for dasatinib and 400 mg (range 129–801) for imatinib. Additional treatment exposure information can be found in Supplementary Table S1.

**Table 2:** Patient disposition of treated patients.

	Dasatinib (*n* = 171)	Imatinib (*n* = 86)	Total (*N* = 257)
Continuing on treatment	135 (79)	68 (79)	203 (79)
Crossed over to dasatinib	–	45 (52)	45 (18)
Imatinib failure	–	44 (51)	44 (17)
Suboptimal response	–	1 (1)	1 (<1)
Not continuing on treatment	36 (21)	18 (21)	54 (21)
Disease progression	5 (3)	1 (1)	6 (2)
Study drug toxicity	12 (7)	4 (5)	16 (6)
Death	1 (1)	2 (2)	3 (1)
Other	18 (11)	11 (13)	29 (11)
Continuing in the study	23 (13)	13 (15)	36 (14)
Not continuing in the study	13 (8)	5 (6)	18 (7)
Withdrew consent	2 (1)	0	2 (1)
Death	3 (2)	4 (5)	7 (3)
Lost to follow-up	1 (1)	0	1 (<1)
Other	7 (4)	1 (1)	8 (3)

Values are *n* (%).

Of the 86 patients randomized to imatinib, 45 (52%) subsequently crossed over to dasatinib, with an overall median time to dasatinib crossover of 9 months (95% CI 6–12) after randomization (Supplementary Fig. S1). Overall, 44 of 86 patients randomized to imatinib (51%) experienced treatment failure, and one patient (1%) had suboptimal response to imatinib. Patient characteristics were consistent between patients who crossed over to dasatinib and those who remained on imatinib after randomization.

The median treatment duration was longer for patients randomized to dasatinib (33 months, range <1–63) vs. imatinib (20 months, range 1–57). For patients receiving dasatinib after crossing over from imatinib (*n* = 45), the median treatment duration on dasatinib was 23 months (range <1–48). For patients receiving imatinib who did not crossover to dasatinib (*n* = 41), the median treatment duration was 33 months (range 1–57). Dose interruptions, escalations, and reductions were experienced by 75 (44%), 23 (13%), and 10 (6%) patients randomized to dasatinib and 37 (43%), 10 (12%), and 7 (8%) patients randomized to imatinib, respectively. To date, 54 (21%) patients discontinued treatment; 12 (7%) and 4 (5%) patients randomized to dasatinib and imatinib, respectively, discontinued due to toxicity. The most common reasons for discontinuing dasatinib due to toxicity were hematological toxicity in six patients (grade 3–4) and pleural effusion in five patients (grade 2–3). Among patients randomized to imatinib, the most common reason for discontinuation due to toxicity was non-hematological toxicity in three patients (all grade 2).

### Efficacy

**Fig. 2:**
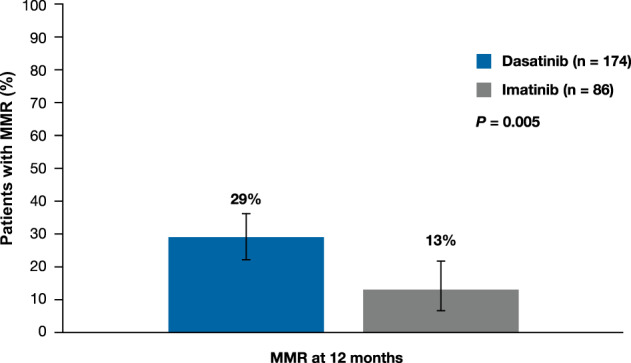
MMR at 12 months in the ITT population (primary endpoint). Error bars represent 95% CI. CI confidence interval, ITT intent-to-treat, MMR major molecular response.

**Fig. 3:**
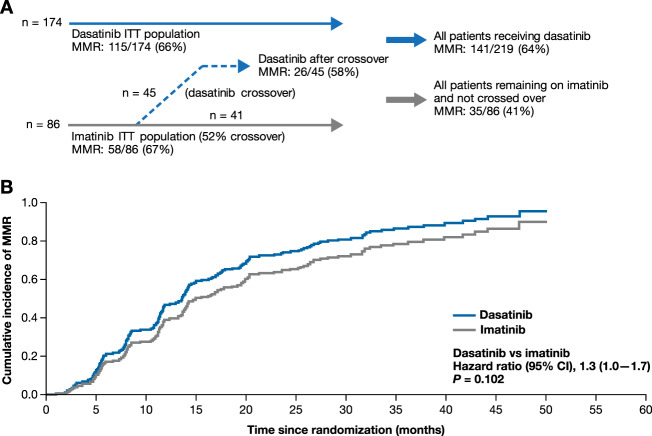
MMR after a minimum follow-up of 24 months. **a** MMR according to study population and crossover. **b** Cumulative incidence of MMR accounting for competing risk. CI confidence interval, ITT intent-to-treat, MMR major molecular response.

The primary endpoint of MMR rate after 12 months was met, with a significantly higher MMR rate in the dasatinib vs. imatinib arm (29% vs. 13%, *P* = 0.005, Fig. 2). Further efficacy analyses described below were performed at a 2-year data cut. Overall, per ITT, 66% of patients randomized to dasatinib and 67% of patients randomized to imatinib achieved MMR (Fig. 3a). When accounting for competing risk, the cumulative incidence of MMR was higher in patients in the dasatinib arm than the imatinib arm (Fig. 3b); however, this finding was not statistically significant as these results were influenced by 30% of patients from the imatinib arm achieving MMR after crossing over to dasatinib. After taking this treatment crossover into account, 141 of 219 patients (64%) on dasatinib, including 115 (66%) initially randomized patients and 26 (58%) patients who crossed over from imatinib to dasatinib, achieved MMR by 24 months. When patients with treatment failure were censored at crossover, MMR was achieved in 35 of 86 patients (41%) on imatinib. Median time to MMR was 14 months (95% CI 12–18) in patients randomized to dasatinib vs. 20 months (95% CI 14–26) in patients who remained on imatinib (*P* = 0.130). In patients initially randomized to imatinib who crossed over to dasatinib, the median time to MMR was 19 months (95% CI 8–38). Cumulatively, 36 patients (21%) on dasatinib and 18 patients (21%) on imatinib, regardless of crossover status, achieved MR^4.5^ by month 24. Patients randomized to dasatinib or imatinib who did not experience treatment failure had similar declines in *BCR-ABL1* transcript levels over time (Supplementary Table S2). The decline in *BCR-ABL1* transcript levels was delayed in patients with suboptimal response to imatinib until crossover to dasatinib. Cumulatively, 147 (85%) patients randomized to dasatinib and 71 (83%) patients randomized to imatinib achieved CCyR. Notably, 29 of the 45 (64%) patients randomized to imatinib who experienced treatment failure achieved CCyR after crossover to dasatinib.

**Fig. 4:**
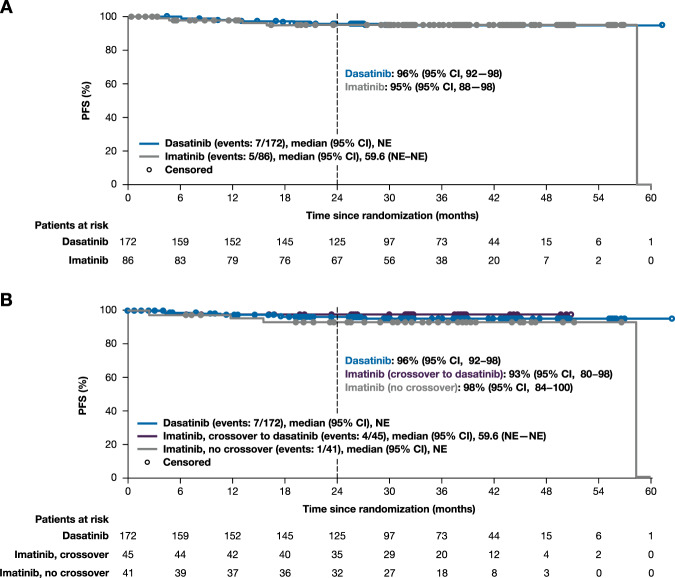
PFS. Kaplan–Meier estimate of PFS in ITT population (**a**) and by switch status (**b**). PFS was defined as the time from randomization to transformation to CML-AP/BC or death, whichever occurred first. All patients who discontinued study treatment were followed for progression and survival unless consent was withdrawn. CI confidence interval, ITT intent-to-treat, NE not evaluable, PFS progression-free survival.

**Fig. 5:**
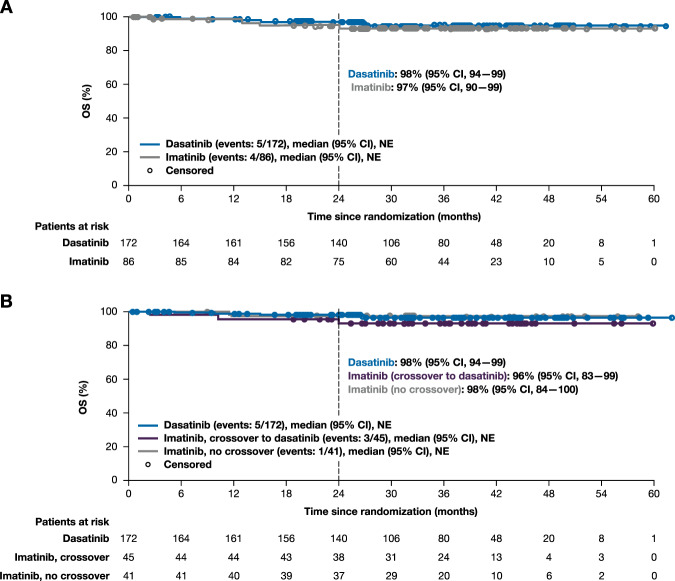
OS. Kaplan–Meier estimate of OS in ITT population (**a**) and by switch status (**b**). CI confidence interval, ITT intent-to-treat, NE not evaluable, OS overall survival.

In the ITT population, PFS at 24 months was 96% (95% CI 92–98) for patients randomized to dasatinib and 95% (95% CI 88–98) for patients randomized to imatinib (Fig. 4a). According to switch status, PFS at 24 months was 96% (95% CI 92–98) in patients initially randomized to dasatinib (early switch), 93% (95% CI 80–98) in patients initially randomized to imatinib who subsequently crossed over to dasatinib, and 98% (95% CI 84–100) in patients randomized to imatinib and without subsequent crossover (Fig. 4b). In the ITT population, OS at 24 months was 98% (95% CI 94–99) in patients randomized to dasatinib and 97% (95% CI 90–99) in patients randomized to imatinib (Fig. 5a). By switch status, 24-month OS was 98% (95% CI 94–99) in patients randomized to dasatinib (early switch), 96% (95% CI 83–99) in patients initially randomized to imatinib who later crossed over to dasatinib, and 98% (95% CI 84–100) in patients receiving imatinib without crossover (Fig. 5b). Six patients in the dasatinib arm progressed to CML-AP/BC, two of whom progressed after discontinuing dasatinib—one discontinued due to hematologic toxicity (progressed 38 days after last dose) and one discontinued due to treatment failure (progressed 11 months after last dose). One patient in the imatinib arm progressed at month 4, and two patients who crossed over to dasatinib progressed—one crossed over at month 12 (progressed at month 15) and one crossed over at month 24 (progressed at month 59).

### Safety

Treatment-related AEs of any grade occurred in 141 (82%) patients randomized to dasatinib and 67 (78%) randomized to imatinib (Table 3). Grade 3/4 treatment-related AEs occurred in 60 (35%) and 36 (42%) patients in the dasatinib and imatinib arms, respectively. Any grade treatment-related AEs were reported in 38 of the 45 patients (84%) who crossed over to dasatinib from imatinib and in 29 of the 41 patients (71%) who remained on imatinib. Serious AEs (of any grade and cause) occurred in 33 (19%) patients on dasatinib and 16 (19%) patients on imatinib, including in eight (18%) patients who crossed over to dasatinib and eight (20%) patients on imatinib without crossover. Pleural effusion occurred in 15 (9%) patients on dasatinib (grade 3/4 severity in three patients), including in five patients (all grade 2–3) who discontinued due to study drug toxicity. In addition, five (11%) patients initially randomized to imatinib who crossed over to dasatinib developed pleural effusion (two cases were grade 3/4). The most common treatment-related non-hematologic AE was headache in patients randomized to dasatinib (15%) and hypophosphatemia in patients randomized to imatinib (13%, Table 3). Grade 3/4 headaches occurred in three (2%) patients in the dasatinib arm, and grade 3/4 hypophosphatemia was observed in five (6%) patients in the imatinib arm. In patients receiving imatinib who crossed over to dasatinib, headache was the most frequently observed treatment-related AE (13%). Treatment-related hematologic toxicity was comparable between both treatment arms, with grade 3/4 neutropenia occurring in 21 (12%) patients on dasatinib, 14 (16%) patients on imatinib, and in 13 (29%) patients on dasatinib after crossover (Table 3). The occurrence of anemia, thrombocytopenia, and leukopenia was also similar across treatment arms. Arterial occlusive events occurred in two patients treated with dasatinib (cardiac angina and cerebral ischemia) and in one patient treated with imatinib who crossed over to dasatinib (ischemic stroke). In total, nine (4%) patients died: five (3%) were randomized to dasatinib and four (5%) to imatinib (three of whom subsequently crossed over to dasatinib). Of the nine deaths, three were due to disease progression (one patient randomized to dasatinib, two patients randomized to imatinib, both of whom crossed over to dasatinib) and one due to study drug toxicity (imatinib with crossover to dasatinib).

**Table 3:** Any grade and grade 3/4 treatment-related AEs reported in ≥5% of all randomized patients in either arm.

Patients with AEs	Patients randomized to dasatinib (*n* = 171)		Patients randomized to imatinib (*n* = 86)		Patients on dasatinib after crossing over from imatinib (*n* = 45)		Patients on imatinib with no crossover to dasatinib (*n* = 41)	
	Any grade	Grade 3–4	Any grade	Grade 3–4	Any grade	Grade 3–4	Any grade	Grade 3–4
Total patients with an AE	141 (82)	60 (35)	67 (78)	36 (42)	38 (84)	25 (56)	29 (71)	11 (27)
Non-hematologic AEs								
Headache	26 (15)	3 (2)	9 (10)	0	6 (13)	0	3 (7)	0
Diarrhea	16 (9)	0	8 (9)	0	4 (9)	0	4 (10)	0
Hypophosphatemia	16 (9)	2 (1)	11 (13)	5 (6)	5 (11)	1 (2)	6 (15)	4 (10)
Pleural effusion	15 (9)	3 (2)	5 (6)	2 (2)	5 (11)	2 (4)	0	0
Rash	14 (8)	0	8 (9)	1 (1)	2 (4)	0	6 (15)	1 (2)
Nausea	13 (8)	0	8 (9)	0	2 (4)	0	6 (15)	0
URT infections	10 (6)	0	3 (3)	0	3 (7)	0	0	0
Asthenia	8 (5)	0	3 (3)	0	2 (4)	0	1 (2)	0
Dizziness	8 (5)	0	2 (2)	0	2 (4)	0	0	0
Pain in extremity	6 (4)	0	6 (7)	0	2 (4)	0	4 (10)	0
Upper abdominal pain	5 (3)	0	4 (5)	0	3 (7)	0	1 (2)	0
Vomiting	4 (2)	0	4 (5)	0	2 (4)	0	2 (5)	0
Fatigue	4 (2)	0	6 (7)	0	3 (7)	0	3 (7)	0
Eyelid edema	2 (1)	0	8 (9)	0	4 (9)	0	4 (10)	0
Hypocalcemia	2 (1)	0	6 (7)	0	4 (9)	0	2 (5)	0
Muscle spasms	2 (1)	0	8 (9)	0	1 (2)	0	7 (17)	0
Hematologic AEs								
Neutropenia	37 (22)	21 (12)	25 (29)	14 (16)	19 (42)	13 (29)	6 (15)	1 (2)
Anemia	39 (23)	11 (6)	21 (24)	3 (3)	13 (29)	3 (7)	8 (20)	0
Thrombocytopenia	39 (23)	18 (11)	15 (17)	9 (10)	13 (29)	7 (16)	2 (5)	2 (5)
Leukopenia	14 (8)	2 (1)	11 (13)	2 (2)	7 (16)	1 (2)	4 (10)	1 (2)

Values are *n* (%).

*AE* adverse event, *URT* upper respiratory tract.

## Discussion

Achievement of EMR may increase the likelihood of attaining a subsequent DMR and having favorable long-term outcomes, but it is not known whether patients without an EMR at 3 months will benefit from an early switch to a potent second-generation TKI. DASCERN is the first prospective trial to demonstrate the potential benefit of early switching to dasatinib in patients without EMR after 3 months of imatinib treatment. In this study, patients who switched to dasatinib at 3 months had a significantly higher MMR rate at 12 months than patients who remained on imatinib (29% vs. 13%, *P* = 0.005), and cumulatively, by month 24 more patients on dasatinib had achieved MMR (64% vs. 41%) once treatment crossover was accounted for. Cumulative incidence of CCyR was similar in the dasatinib and imatinib arms; however, 29 (64%) patients initially randomized to the imatinib arm achieved a CCyR after having a suboptimal response and crossing over to dasatinib. Overall, these findings support the need for early monitoring and intervention for newly diagnosed patients with CML-CP not receiving a second-generation TKI as first-line therapy, and indicate that patients who fail to achieve EMR with first-line imatinib benefit from switching to dasatinib at 3 months.

Previous studies have shown that early intervention may be considered when patients have suboptimal cytogenetic responses on first-line imatinib [15–20]. Quintas-Cardama et al. retrospectively demonstrated that response rates and survival were most favorable when dasatinib was administered early after imatinib failure, with 72% of patients who received dasatinib after loss of major cytogenetic response (MCyR) to imatinib achieving CCyR, compared with 42% of patients who were treated after loss of both MCyR and CHR [15]. In the same study, event-free survival (EFS) was higher after earlier vs. later dasatinib intervention; the EFS with early intervention was in line with previous reports for second-line imatinib after interferon failure [15, 16]. In the TIDEL (Therapeutic Intensification in De Novo Leukaemia)-II study, patients who started on imatinib and switched to nilotinib due to intolerance, treatment failure, or loss of response, achieved improved survival outcomes (including OS and transformation-free survival), although only a small number of patients (*n* = 54) switched to nilotinib, and the study included an assessment of imatinib plasma trough levels, which are not routinely assessed in clinical practice [17]. In the LASOR (Imatinib Dose Optimization vs. Nilotinib in CML Patients With Suboptimal Response to Imatinib) trial, patients with suboptimal cytogenetic responses to imatinib were more likely to achieve CCyR and MMR after switching to nilotinib, although the difference was not statistically significant [18]. It has also been shown that imatinib dose escalation fails to “rescue” those patients with suboptimal responses [19]. Despite these observations, there has been a general lack of larger, prospective studies exploring the significance of early intervention for patients with suboptimal molecular responses to first-line imatinib.

Attainment of improved and rapid molecular responses with TKI therapy could help decrease the probability of transformation and improve long-term outcomes. It has been demonstrated that patients treated with first-line imatinib who did not achieve an EMR had significantly lower 8-year probabilities of OS (57% vs. 93%), PFS, and complete molecular response than patients who achieved EMR at 3 months [21]. Consequently, achieving EMR at the 3-month molecular milestone is considered an optimal response [1]. More patients treated with second-generation TKIs achieve these treatment goals compared with those treated with imatinib [2, 3]. In the ENESTnd study (Study of Imatinib vs. Nilotinib in Adult Patients With Newly Diagnosed Philadelphia Chromosome Positive Chronic Myelogenous Leukemia in Chronic Phase), 89–91% of patients who received nilotinib achieved EMR at 3 months, compared with 67% of patients who received imatinib [3]. Similarly, in the DASISION study, 84% of patients achieved EMR at 3 months with dasatinib vs. 64% with imatinib [2]. Furthermore, patients who achieved EMR with dasatinib had improved 5-year OS and PFS rates, as well as reduced rates of transformation [2].

In DASCERN, no differences in OS and PFS outcomes were observed between the treatment arms—a finding that was likely influenced by the short follow-up period of this study. However, an extended follow-up will be of interest as the crossover from the imatinib arm is expected to have an impact on the differences in long-term outcomes. Interestingly, PFS in DASCERN appears higher (96% [95% CI 92–98]) than has been previously reported for dasatinib [2]. The seemingly favorable PFS may be related to the study definition of progression (transformation to CML-AP/BC or death from any cause since randomization), which differs from the definition of progression historically used in clinical trials with dasatinib. For example, progression was defined in DASISION as loss of CHR, MCyR, transformation to CML-AP/BC, death, or increasing white blood cell counts [2, 22]. In addition, patients who had progressed before 3 months were not eligible for this trial. Excluding these patients (although few) from the PFS calculation may also influence the overall PFS rate. Furthermore, ~50% of patients who were randomized to remain on imatinib experienced treatment failure and required crossover to dasatinib at a later time. In this subgroup of patients (later switch/crossover), a trend toward worse PFS was observed compared with those who were randomized to dasatinib at study entry (i.e., 3 months after start of imatinib; early switch), suggesting that a delayed treatment switch may have increased the risk of transformation or death in this patient population. Notably, a previous long-term follow-up study has shown that most progression events occur within the first 3 years of imatinib treatment [23]. These observations highlight the need for early monitoring and intervention in patients with suboptimal responses to imatinib.

The early switch to dasatinib in DASCERN did not increase the incidence of treatment-related events. In addition, the rates of treatment-related hematologic AEs in those who switched to dasatinib and in those who remained on imatinib were similar. Interestingly, the incidence of pleural effusion in this study (9%) was lower than that seen in other dasatinib studies [24, 25]. Indeed, in a 2-year follow-up of the DASISION study, pleural effusion was observed in 14.3% of patients, with a discontinuation rate of 1.9% [24]. A similar rate (14%) was observed in the phase 3 dasatinib dose optimization study (CA180–034), in which most cases were managed with temporary dose interruption or reduction; only three (1.4%) patients required dasatinib discontinuation due to pleural effusion [25]. The lower incidence of pleural effusion in DASCERN may be due to the relatively young age of the patients, as younger patients have been reported to be at a reduced risk of developing pleural effusion after initiating dasatinib therapy [26]. However, a longer follow-up is required as pleural effusions may occur later in the course of therapy with dasatinib.

In this first prospective randomized study to explore the benefit of early switching to dasatinib, the greater response rates with dasatinib and the observation that approximately half of patients who did not achieve EMR with imatinib subsequently met treatment failure criteria and crossed over to the dasatinib arm, provide further support for using strategies that increase the probability of achieving optimal responses early on in the treatment paradigm. As MMR rates are known to improve with longer treatment duration [27], additional follow-up will help to determine if the rates continue to favor the use of dasatinib and whether this early benefit translates into a greater probability of achieving DMR and improved PFS and OS. In summary, initial findings from DASCERN provide new insight into the potential benefit of switching to dasatinib in patients failing to achieve important treatment milestones with first-line imatinib. Furthemore, these data support the importance of early monitoring for patients who do not receive a second-generation TKI as their first-line treatment, and suggest that pre-emptive switching to dasatinib in such instances may provide clinical benefit.

### Supplementary information

Supplementary information
