# Supplementary material for: Dasatinib vs. imatinib in patients with chronic myeloid leukemia in chronic phase (CML-CP) who have not achieved an optimal response to 3 months of imatinib therapy: the DASCERN randomized study
Source: Leukemia. 2020 Apr 7;34(8):2064–73. doi: 10.1038/s41375-020-0805-1 (PMC7387297; doi:10.1038/s41375-020-0805-1)
Supplement: Supplementary file 1 — Supplementary information [file 41375_2020_805_MOESM1_ESM.docx]

# Supplementary information

##### Fig. S1 CONSORT diagram

*Most common reasons for failure: inadequate molecular response (n = 21; defined as *BCR-ABL1* > 10% and/or no partial cytogenetic response [PCyR; Ph+ > 35%] at 6 months, or *BCR-ABL1* > 1% and/or PCyR [Ph+ > 0%] at 12 months), loss of CHR or CCyR (n = 10), other (n = 6), loss of MMR (n = 3), *BCR-ABL1* mutations poorly sensitive to imatinib (n = 2), absence of cytogenetic response (CyR; Ph+ > 95%) at 6 months (n = 1), and response less than CCyR at 18 months (n = 1)

**
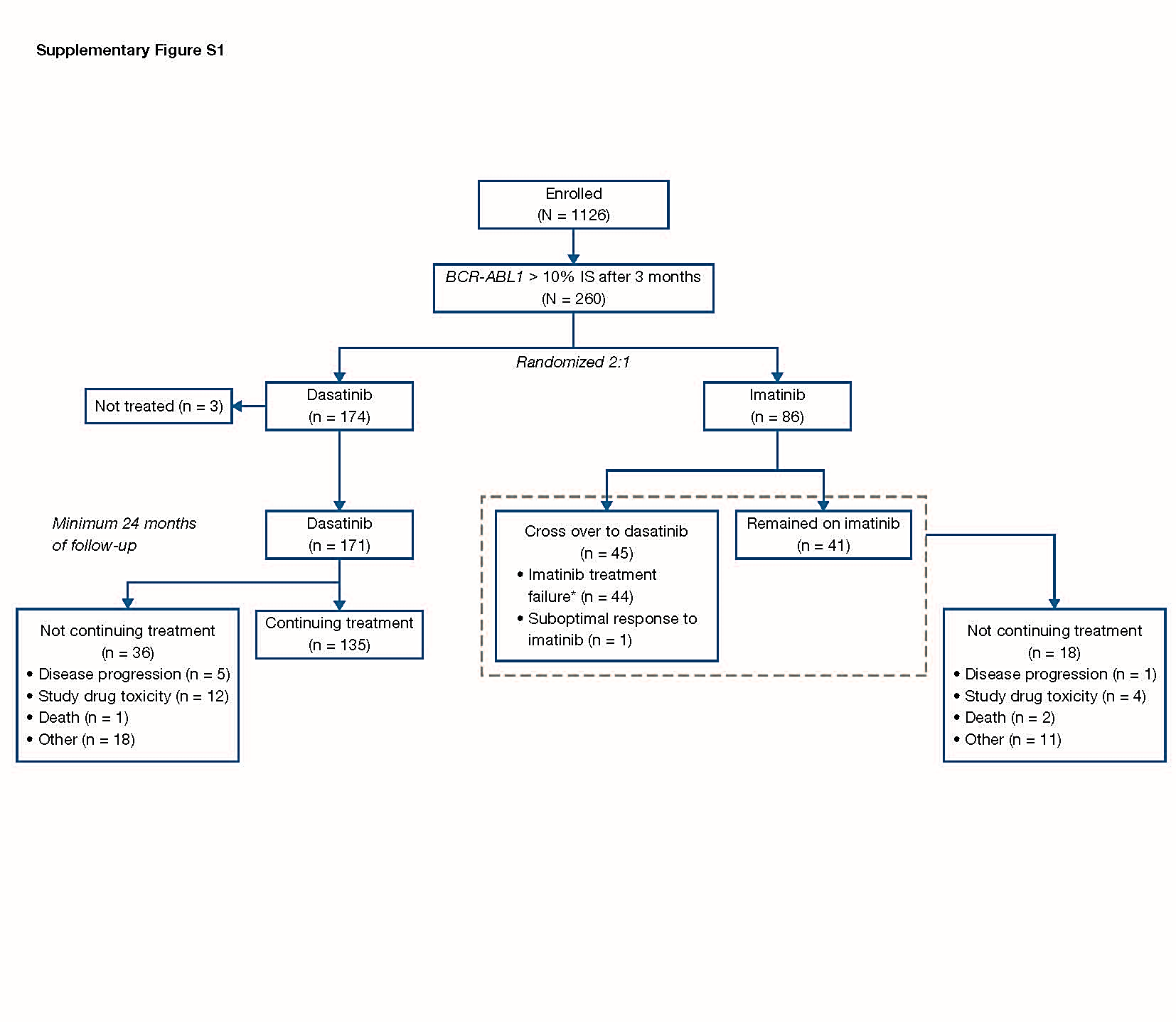
**

##### Supplementary Table S1 Treatment exposure

|  | **Patients randomized**  **to dasatinib**  **(n = 171)*** | **Patients randomized**  **to imatinib**  **(n = 86)** | **Patients on dasatinib after crossing over from imatinib**  **(n = 45)** | **Patients on imatinib with no crossover to dasatinib**  **(n = 41)** |
| --- | --- | --- | --- | --- |
| Daily dose, median (range), mg | 100 (26–136) | 400 (129–801) | 97 (45–600) | 400 (129–801) |
| Treatment duration, median (range), months | 33 (< 1–63) | 20 (< 1–57) | 23 (< 1–48) | 33 (1–57) |
| Patients with ≥ 1 dose interruption | 75 (44) | 37 (43) | 21 (47) | 14 (34) |
| Patients with ≥ 1 dose escalation | 23 (13) | 10 (12) | 2 (4) | 4 (10) |
| Patients with ≥ 1 dose reduction | 10 (6) | 7 (8) | 4 (9) | 3 (7) |

Values are n (%) unless otherwise noted.

*****Three patients randomized to dasatinib decided to withdraw their consent prior to medication intake and were not included

##### Supplementary Table S2 *BCR-ABL1* transcript levels over time

|  | **Patients randomized to dasatinib (regardless of response)**  **(n = 172)** | **Patients on imatinib who crossed over to dasatinib (due to imatinib failure)**  **(n = 45)** | **Patients on imatinib with no crossover to dasatinib***  **(n = 41)** |
| --- | --- | --- | --- |
| Median *BCR-ABL1* transcript levels (interquartile range), %  Baseline  Month 6  Month 12  Month 24 | 23.3 (16.2–33.8)  1.9 (0.7–8.8)  0.3 (0.1–0.8)  0.1 (0–0.2) | 24.8 (15.0–38.4)  9.9 (2.9–16.0)  2.5 (0.6–14.3)  0.4 (0.1–3.7) | 17.7 (12.1–24.8)  2.1 (0.9–3.4)  0.2 (0.1–0.5)  0.1 (0–0.1) |

*****Optimal responders
